# Self-compassion Education for Health Professionals (Nurses and Midwives): Protocol for a Sequential Explanatory Mixed Methods Study

**DOI:** 10.2196/34372

**Published:** 2022-01-13

**Authors:** Mary Steen, Shwikar Mahmoud Etman Othman, Annette Briley, Rachael Vernon, Steven Hutchinson, Susan Dyer

**Affiliations:** 1 Nursing, Midwifery and Health Northumbria University Newcastle United Kingdom; 2 UniSA Clinical and Health Sciences University of South Australia Adelaide Australia; 3 Faculty of Nursing South Valley University Qena Egypt; 4 College of Nursing & Health Sciences Flinders University Adelaide Australia; 5 SA Prison Health Service, Central Adelaide Local Health Network SA Health Adelaide Australia; 6 Nursing & Midwifery Clinical Practice Development Unit Women's & Children's Health Network Adelaide Australia

**Keywords:** self-compassion, mixed methods research, study protocol, health professionals, nurses, midwives

## Abstract

**Background:**

A few recent studies have reported that having the ability to provide self-compassion can reduce health professionals’ levels of anxiety and stress, the risk of compassion fatigue, and burnout, and it can generally improve their well-being. Therefore, there is evidence to support further research into the investigation and exploration of self-compassion education and training for health professionals.

**Objective:**

This study aims to increase the knowledge and understanding of self-compassion and how this may enhance the health and well-being of health professionals.

**Methods:**

The proposed research study will adopt a sequential explanatory mixed methods design. This study will be conducted in 3 phases. Phase 1 will use a pre-educational self-compassion questionnaire (web-based survey) to collect data from participants at 3 time points (before, immediately after, and after follow-up at 6-8 weeks) after they have attended a self-compassion education and training program. Phase 2 will use an interview schedule to explore the participants’ views and experiences through a follow-up focus group or individual interview. Finally, phase 3 will include data integration and dissemination of key findings and recommendations.

**Results:**

This study was approved by the Women’s and Children’s Health Network Human Research Ethics Committee and the Human Research Ethics Committee at the University of South Australia on June 26, 2021 (ID: 204,074). A scoping review was conducted to inform this research study (focusing on nurses and midwives). The preparatory phase was completed in April 2021. Phase 1 is expected to be completed by June 2022 and phase 2 will commence in July 2022.

**Conclusions:**

The key findings from the data integration for this research project will provide in-depth details and insights to broaden the discussion about self-compassion and its influence on health professionals’ health and well-being. Health professionals (nurses and midwives) may benefit from self-compassion education and training programs to improve their health and well-being.

**International Registered Report Identifier (IRRID):**

PRR1-10.2196/34372

## Introduction

### Background

Self-care relates to any activity undertaken to take care of oneself and encompasses physical, spiritual, emotional, and mental health. Self-care is well recognized to reduce levels of anxiety and stress and thus improve mood. Unfortunately, self-care is often neglected, and being kind and compassionate to oneself is overlooked. However, self-compassion has been embedded in Buddhist philosophy and meditation and has been practiced for over 2500 years. Self-compassion was defined as “being caring and compassionate towards oneself in the face of hardship or perceived inadequacy,” [1]. Neff describes three interrelated elements of self-compassion: self-kindness, common humanity, and mindfulness [2]. Self-kindness involves warmth and an understanding for oneself when faced with difficulties in life and painful experiences and not being overly critical and judgmental of oneself. Common humanity involves recognizing that difficulties in life and painful experiences do not just happen to you but are a shared human experience. Mindfulness involves taking a balanced approach for negative emotions and neither suppressing or exaggerating these and a willingness to acknowledge these negative emotions with openness and clarity (mindfulness-awareness). Self-compassion is related to overall psychological well-being [3,4].

There is some evidence that when a person has high levels of anxiety and stress and history of depression, this is associated with low levels of self-compassion [2,5]. However, much of the research that has been undertaken has involved students as the population of interest and, more recently, military veterans [6]. It has been discussed that when a person has the ability to have self-compassion, they are more inclined to have good interpersonal relationships and experience a greater sense of self-worth and happiness compared with a person who has an impairment in self-compassion [7]. However, there appears to be limited research examining and exploring what self-compassion as a component of self-care is and its relationship with health and well-being for health professionals. Therefore, there is justification to undertake research to investigate and explore what self-compassion as a component of self-care means and the impact of education and training on health professionals. In this study (*You Matter: Finding Your Self-Compassion for health professionals’ education*), the first group of participating health professionals will be midwives and nurses.

### Significance of Self-compassion for Health Professionals

A few recent studies have reported that self-compassion can reduce the levels of anxiety and stress, risk of compassion fatigue, and burnout and can generally improve well-being [8-11]. For example, a cross-sectional study involving primary health care professionals was undertaken to assess the impact of self-compassion as a protective factor against burnout [10]. This study reported that low levels of self-compassion increase the susceptibility to burnout among primary health care professionals. In addition, self-compassion has been shown to increase health professionals’ ability to manage their negative emotions and prevent negative consequences, such as burnout, compassion fatigue, and depression, when undertaking their roles and responsibilities [12].

Self-compassion has also been reported to be associated with sleep patterns and resilience; a study that included dieticians, nurses, physicians, social workers, and others showed that the quality of sleep and resilience were strongly correlated with both self-compassion and mindfulness [13]. This study reported that sleep disturbances were strongly correlated with perceived stress and poorer health, but this was reduced when mindfulness and self-compassion were practiced. Similar findings were reported for resilience and less stress, and improved mental health was associated with practicing mindfulness and self-compassion.

A study conducted in the United States recruited a diverse range of health professionals, that is, nurses, physicians, and social workers, who were asked to participate in three web-based modules of education and training that included: (1) gratitude, (2) positive words, and (3) loving-kindness and compassion meditation [14]. The findings showed that this education or training was associated with statistically significant improvements in gratitude, well-being, self-compassion, and confidence in providing compassionate care. Therefore, web-based education and training appear beneficial to a diverse range of health professionals but further research is required to confirm or refute these findings. Health professionals often work in highly demanding environments and situations and are also part of the community at large; therefore, they are exposed to both professional and personal stressors. Self-compassion education and training may improve awareness and increase the health professionals’ ability to have self-compassion, which may act as a buffer against poor mental health and maintain their well-being and the ability to be compassionate with others.

### Findings and Conclusions From a Scoping Review

A scoping review was conducted to assess the influence of self-compassion on midwives and nurses, and it reported that self-compassion appears to help reduce work-based stressors such as anxiety, compassion fatigue, and burnout in nurses and midwives [15]. This review highlighted that there is some evidence suggesting that self-compassion can improve caring efficacy [16], empathy [17], and emotional intelligence in nurses [18]. In addition, self-compassion can provide overall improvement in the midwives’ and nurses’ well-being and ability to provide compassionate care [19]. Positive components of self-compassion (ie, self-kindness, common humanity, and mindfulness) are generally associated with compassion satisfaction, job satisfaction, and better sleep quality in nurses [20]. Therefore, the influence of self-compassion on midwives’ and nurses’ health and well-being may be an important factor that has implications for future self-care strategies.

This scoping review concluded that self-compassion education and training may improve awareness and increase midwives’ and nurses’ ability to have self-compassion. Therefore, there is an urgent need to explore and investigate the influence of self-compassion on these health professionals, particularly midwives, as this review highlighted a lack of studies and that most studies were related to nursing.

### Preparatory Phase

An educational workshop was developed from a literature search relating to self-compassion and health professionals, specifically focusing on midwives and nurses [15,21] and the primary researcher (MS) attending self-compassion education classes.

#### You Matter: Finding Your Self-compassion Workshop

The *You Matter: Finding Your Self-Compassion education workshop* has been piloted with clinical educators. Two workshops were held at the chief nurse’s education rooms in Adelaide on November 3, 2020. Overall, 21 clinical educators who were members of the South Australia Practice Development Network and employed by South Australia Health attended the workshops.

The workshop aimed to increase awareness and ability for self-compassion.

The workshop objectives were as follows: to explore the benefits of self-care, to introduce the five ways to well-being, to demonstrate the links between compassion and self-compassion, to explore the three elements of self-compassion, to discuss the evidence relating to self-compassion, to dispel the myths surrounding self-compassion, and to develop some self-compassion strategies.

#### Workshop Evaluation

##### Before the Workshop

A total of 21 participants completed an assessment form to assess the baseline information. The assessment form included questions about what self-compassion was, what it meant to the participants personally and whether they had received any previous education for self-compassion. The participants were asked to complete the Self-Compassion Scale short version [22]. This scale consists of 12 statements to assess self-compassion. Data analysis of the responses to the 12 statements demonstrated a self-compassion mean score of 38.38 (SD 3.43), which indicates that the clinical educators had a moderate to low level of self-compassion. Therefore, this finding further supports the justification for providing self-compassion education to health professionals and teaching clinical educators to become trainers.

##### After the Workshop

Clinical educators who attended the workshop were invited to complete a workshop evaluation form. This evaluation confirmed that all clinical educators acknowledged the importance and value of compassion for self and others. Almost all the educators strongly agreed that the workshop provided them with a clear understanding of what self-compassion was and the strategies that could be used. The interactive workshop content helped them understand the underpinning philosophy and increased their awareness of the health and well-being benefits of self-compassion. All educators reported that they would practice self-compassion in the future. A total of 10 participants confirmed that they would like to attend a *Train the Trainer* session to teach self-compassion care and strategies to other health professionals. Overall, this workshop met the educators’ expectations at both personal and professional levels. The evaluation following the workshop provided evidence that the aims and objectives of the self-compassion workshop were achieved.

##### Clinical Educators to Become Trainers

The clinical educators who attended the *You Matter: Finding Your Self-Compassion* workshops and expressed an interest in becoming a trainer were given an opportunity to attend a *Train the Trainer* session. As a result, a *Train the Trainer* session was facilitated in July 2021. Two further training session were undertaken in October and November 2021. Approximately 8-10 clinical educators have been trained to facilitate the workshops.

### Research Aim

The overall aim of the study is to increase the knowledge and understanding of self-compassion and how this may enhance health and well-being of health professionals. However, for this first study, we are focusing on health professionals who are nurses and midwives.

### Specific Objectives

The specific objectives of this study are as follows: to find out and explore what self-compassion means to nurses and midwives; to find out what being compassionate to others means to nurses and midwives; to determine if there is an association between self-compassion and the levels of anxiety and stress, mood, and well-being; to provide education to develop self-compassion strategies; and to enhance nurses and midwives’ skills for self-compassion

### Research Questions

This study aims to answer the following research questions: (1) What does self-compassion mean for health professionals (nurses and midwives)? (2) What does compassion for others mean to health professionals (nurses and midwives)? (3) What influence will self-compassion education and training have on health professionals’ (nurses and midwives) health and well-being? (4) Are high levels of anxiety and stress associated with low levels of self-compassion, mood, and well-being among health professionals (nurses and midwives)?

## Methods

### Study Design

This proposed research will use a mixed methods approach and undertaken in two stages: quantitative phase and qualitative phase.

A sequential explanatory mixed methods study will be conducted to investigate and explore the influence of self-compassion on health professionals’ health and well-being. The sequential explanatory mixed methods study design was chosen to combine the strengths of both quantitative and qualitative research methods to answer the research questions and meet this study’s aims and objectives. This mixed methods approach will be guided and underpinned by a pragmatist worldview that focuses on a research problem and on how to answer a research question and address the aims and objectives of a research study [23]. Pragmatism uses an integrative philosophy that combines quantitative and qualitative research without restrictive methodological directions. This mixed

methods study provides a flexible and transparent approach to unexpected data findings [24]. In addition, the mixed methods approach will provide in-depth details and insights to broaden the discussion and strengthen the study’s findings to answer the research questions, draw key conclusions, and identify further research areas [25]. The planned time to undertake this mixed methods study involving midwives and nurses will be 12 months. Further research is planned to involve other disciplines of health professionals on the completion of this initial study.

### Conceptual Framework

A conceptual framework for this mixed methods study will include a philosophical stance and strategies that will underpin and guide the specific direction through which the research will be undertaken.

### Philosophical Assumptions and Pragmatism

It is important to determine the most suitable philosophical assumptions for the mixed methods study, as it is considered the main foundation for research [26]. Pragmatism was chosen because this stance supports the use of both qualitative and quantitative research methods [27], and it appears to be the most appropriate philosophy for a mixed methods approach [26,28,29].

Pragmatism accepts the views of both postpositivists and interpretivists by using an integrative logic, linking and combining quantitative and qualitative approaches [23]. It uses an integrative philosophy that combines both quantitative and qualitative research without restrictive methodological directions. Therefore, these combinations of methods and ideas provide the best conceptual framework to address and provide reasonable answers to research questions through a mixed methods approach [26].

### Strategies for a Sequential Explanatory Mixed Methods Design

This research study will adopt a sequential explanatory mixed method design, which consists of gathering data separately and sequentially into 2 phases [23]. Phase 1 involves quantitative data collection and analysis. Phase 2 involves qualitative data collection and analysis. The findings from these 2 phases will be combined and mixed for integration and final analysis. The adopted sequential explanatory design will be the best to answer the proposed research questions and draw broader conclusions. Phase 3 will be undertaken on completion of the data collection and analysis and will involve data integration, dissemination, and translation of findings and recommendations.

### Methods to Describe Data Collection

Phase 1 will use a pre-educational self-compassion questionnaire (web-based survey), which includes 4 measurement scales. This questionnaire will be used to collect data from participants at 3 time points (before, immediately after, and after follow-up at 6-8 weeks) after attending the self-compassion education and training program. After collecting the baseline data from participants regarding their compassion, well-being, anxiety and stress, and mood, they will be invited to attend a self-compassion workshop. Participants will then be reminded to submit an immediate questionnaire and a follow-up questionnaire 6 to 8 weeks after attending the self-compassion workshop. Phase 2 will use an interview schedule to explore the participants’ views and experiences. Participants will be invited to participate in either a follow-up focus group or individual interview (depending on COVID-19 restrictions) via the study website.

### Phase 1: Quantitative Phase (Workshop Education and Evaluation)

This phase will include a web-based survey, a self-compassion workshop, an immediate evaluation, and a follow-up evaluation of the participants after 6 to 8 weeks of attending the self-compassion education and training program.

#### Aims and Objectives of the Web-Based Survey

The aim of the You Matter: Finding Your Self-compassion for Health Professionals (Nurses and Midwives) web-based survey (3 time point questionnaires) is to find out what being compassionate to oneself means to health professionals (nurses and midwives) and its influence on their health and well-being.

The primary objectives are to investigate and explore what self-compassion means and measure levels of self-compassion and well-being for registered health professionals with the Australian Health Practitioner Regulation Agency in South Australia (nurses, midwives) and to assess whether there is an association between self-compassion and levels of anxiety and stress, mood, and well-being among the health professionals (nurses and midwives).

#### Recruitment and Sample Size

Health professionals (nurses and midwives) residing and employed in South Australia will be invited to participate in the study (see Textbox 1 for the inclusion and exclusion criteria). An invitation will be sent via South Australia Practice Development Network, professional bodies, and social media outlets such as Facebook (Meta Platforms, Inc) and Twitter (Twitter, Inc). A dedicated study website with the domain name “https://compassionselfcare.org/” is being designed and will be used for all nurses and midwives to access and participate in the study [30]. In addition, advertisements will be distributed through local health networks in South Australia, including the following: Central Adelaide Local Health Network; Northern Adelaide Local Health Network (Lyell McEwin Hospital); Southern Adelaide Local Health Network (Flinders Medical Centre); Women’s and Children’s Health Networks (Women’s and Children’s Hospital); and Riverland Mallee Coorong Local Health Network.

**Inclusion and exclusion criteria for the study.**

**Inclusion criteria**

- Qualified and registered health professionals with Australian Health Practitioner Regulation Agency working in the following roles:
  - Registered midwives
  - Registered nurses
- Residing and working in South Australia

**Exclusion criteria**

- Not qualified and currently not registered health professionals
- Working outside South Australia

#### Participants

A nonprobability convenience sample will be used in this study. Recruitment will commence with nurses and midwives as the first cohort of participants. Other health professionals registered with the Australian Health Practitioner Regulation Agency will be recruited later as the research project progresses, for which further ethics approval will be sought.

In South Australia, there are approximately 57,784 registered health practitioners as per the 2018-2019 registration statistics; of these, 678 (1.17%) are midwives, 32,361 (56%) are nurses, and 1854 (3.21%) have dual registration. A power calculation using a single-factor, repeated measures design estimated that a sample of 380 participants, measured at 3 time points, will achieve 95% power to detect differences before and after education using a Geisser-Greenhouse corrected *F* test at a .05 significance level (*P*=.05). Therefore, the study aims to recruit 400 health care professionals (nurses and midwives) to account for the potential loss to follow-up, with the intervention requiring completion of pre-, immediate, and posttest educational questionnaires.

#### Study Website

A specific study website is currently being designed to include detailed information about the study for nurses and midwives. A home page will introduce the research project and state its aims and objectives. Other pages will provide information related to participation in the study, ethical considerations, the research team, and the web-based questionnaires to complete. In addition, it will include an invitation for nurses and midwives to attend an educational workshop, a follow-up focus group or an individual interview (face-to-face, zoom, or telephone), and the instructions to contact the primary researcher (MS). Additional information will be provided at a later stage for future research involving other health professional disciplines and further workshops as the project progresses (the future studies will be part 2: managing emotions and conflict and part 3: ways to well-being).

#### Web-Based Survey (Questionnaires)

The website will provide a link to a web-based survey for participants to complete once they have clicked on the button expressing their consent to participate in the study. The web-based survey (questionnaires) will be hosted using the REDCap (Research Electronic Data Capture; University of South Australia) software from University of South Australia.

The following measurement scales will be included in the web-based survey:

- The Self-Compassion Scale short version (12-items) scale consists of 12 items related to self-kindness (2, 6 items), self-judgment (11, 12 items), common humanity (5, 10 items), isolation (4, 8 items), mindfulness (3, 7 items), and overidentification (1, 9 items) [22].
- The Warwick and Edinburgh Mental Well-Being Scale short version (7 statements) scale assesses the study population’s mental and emotional well-being and psychological functioning, which describes feelings and thoughts (functions) [31].
- The Capture My Mood Scale—adapted version (5 items scale), with scores ranging from 1 to 5, is used to assess the mental health status [32].
- The State-Trait Anxiety Scale short version (6-items) [33].

The validity and reliability of these 4 scales have been previously assessed.

To determine the association between a health professional’s prior anxiety and stress levels with their levels of self-compassion, a history of anxiety and stress will be assessed by including a specific and open-ended question asking the participating health professionals to provide further details. These questions will include the following: *Have you experienced high levels of anxiety in your life?* *Have you experienced high levels of anxiety when providing health care?* *Have you experienced stress at any time in your life?* *Have you experienced stress while providing health care?*

#### Compassion and Self-care Education and Training Workshop

This workshop will focus on facilitating and evaluating *You Matter: Finding Your Self-Compassion for health professionals*. Furthermore, 2 trained facilitators will run the workshops.

#### Recruitment of Participants

Approximately 400 health professionals (nurses and midwives) who have submitted the web-based survey (prequestionnaire) will be invited to attend workshops at the study sites (University of South Australia and South Australia Health). The workshops will be undertaken for 6 months, and the number of participants will be between 10 and 12 per workshop.

#### Workshop Content and Components

A 3-hour workshop will be conducted and facilitated by 2 facilitators (1 clinical and 1 educator or trainer from University of South Australia). The workshop will cover self-compassion, research and evidence, dispelling myths, communication, self-care, befriending oneself, acknowledging and accepting negative emotions, and being compassionate to oneself. The workshop will also include time for refreshment and for completing an evaluation of the workshop.

The teaching materials will include a workbook, PowerPoint (Microsoft, Inc) slides, brochures, memo cards, and recommended reading materials. Several activities will include interactive group work, personal reflections, self-compassion exercises, and deep relaxation. Health professionals (nurses and midwives) who participate in the workshop will be given a certificate and awarded 3 points toward continuing professional development.

#### Posteducational Workshop Evaluation Questionnaire

The pre-educational workshop questionnaire (web-based measurement scales) will be completed again at the end of the workshop to evaluate the health professionals’ responses after attending the educational workshop. This questionnaire will be provided as a hard copy or a web-based link at the study website. It will include measurement scales for self-compassion, well-being, mood, anxiety, and stress.

#### Follow-up Educational Workshop Evaluation Questionnaire

A member of the research team will contact the participants who attend the self-compassion workshop via telephone or email approximately 6 to 8 weeks after the workshop. The researcher will remind the participants to complete the final posteducational questionnaire (follow-up after test) through the study website to reassess their knowledge and ability for self-compassion. Completing the questionnaires for phase 1 will not take longer than 10 to 15 minutes.

#### Quantitative Data Analysis

Data from the questionnaires (3 time points) will be entered into SPSS version 26 (IBM Corp). Descriptive analysis will examine the participants’ sociodemographic characteristics, such as age, level of education, years of experience, type of occupation and employment, and previous education related to compassion. Inferential data will measure correlations and statistical significance, and the chi-square test will be used to assess categorical data. Cronbach α and Pearson correlation statistical tests will be used to assess high and low levels, respectively, recorded by the measurement scales included in the web-based survey. Repeated measures analysis of variance will be used to examine and compare the differences in the variables before, immediately after, and after follow-up of the workshop (over 3 time points) and to see if self-compassion, well-being, and mood have increased and being maintained. Spearman correlation coefficient tests will be used to evaluate the strength and relationship between 2 or more variables (anxiety, mood, self-compassion, and well-being). A biostatistician will be available to assist with the analysis.

### Phase 2: Qualitative Phase

#### Focus Group and Interview

A subgroup of participants will be invited to either a follow-up focus group or an individual interview at 3 months (in person or web-based, depending on COVID-19 restrictions) after completing the post follow-up questionnaire. Upon completion of the questionnaire, a popup link on the study website will allow the participants to consent to be contacted by a researcher for an interview. Interviews will be conducted in a private room at the University of South Australia, Flinders University, or at one of the study sites.

An interview schedule, including prompts, will be used to guide the undertaking of interviews [34]. The interview guide will be developed from the literature evidence and the findings of phase 1. A purposive sampling technique will be used to recruit and represent health professionals (nurses and midwives) working in South Australia’s metropolitan and rural regions. In addition, a selection criterion will be used to recruit the participants. A minimum of 2 participants will be recruited from different clinical practice areas. The opportunity to attend a focus group or an individual interview will be offered to the nurses and midwives or until data saturation is reached. Data from the participants’ interviews will be recorded and transcribed verbatim. The participants who agree and consent to participate in a follow-up interview will be given an opportunity to cross-check their responses to the questions answered (guided by the interview schedule) at the end of the interview to confirm or refute that the researcher has recorded and interpreted their answers correctly. A summary of the verbatim data will be uploaded to the study website for all participants to access and read.

The transcripts will be analyzed until data saturation is reached and follow-up of participants will then cease. Data saturation is achieved when no new information emerges from the interview data [34-36]. Data saturation can be reached with a few participants (6-12) as well [35,37]. Finally, a thematic analysis will be undertaken and guided by the 6 stages of the thematic framework [38,39].

#### Qualitative Data Analysis

##### Approach Used

The thematic analysis will be used to evaluate the interview data using a reflexive approach. This method is used to identify, analyze, and report patterns or themes within the data. It organizes and describes data sets in detail and can be used to interpret various aspects of the research topic. The six-phase framework for conducting thematic analysis [38,39] will be used as described in the following subsections.

##### Familiarization With Data

The researcher will start listening and relistening to the audio and readings several times to gain depth and breadth of the data related to the topic studied and become immersed and engaged with the data. During this phase, the researcher will start taking notes, marking ideas, and feeling curious.

##### Generating Codes

After becoming familiar with the data, the researcher will take notes and mark ideas, which would mean more details and engagement with the data. It will include focusing on, and making sense of, the data rigorously and systematically. The data will be organized under similar meanings and patterns to develop diverse codes to build themes.

##### Constructing Themes

After the data were coded and collated, the researchers will sort different codes into potential themes and collate all relevant coded data extracts within identified themes using codes. This will be achieved through building blocks or substantial patterns of meaning throughout the data and thematic mapping to visually explore the potential themes and subthemes. Themes should provide a coherent meaning and thread to answer the research question.

##### Revising Themes

The researcher will compile all coded data for each of the main themes and review them to ensure that the data are all connected and related to the main concept, as well as check the theme against the whole data set. This step will focus on an in-depth understanding of each theme’s central concept and boundaries, including subthemes, overarching themes, and the overall theme story.

##### Defining and Naming Themes

In this phase, the researcher will ensure that all themes and themes’ names were clear, concise, and comprehensive to represent the meaning of the whole data and were related to the research question.

##### Producing Reports

A final report will be written and will test how the themes work individually and in relation to the data set and the overarching concept. It will also involve revising the research questions, objectives, and the previous steps of coding and themes to ensure that these themes answer the research question.

### Ethical Considerations

Ethical approval has been granted by the South Australian Health via the web-based research Governance and Ethics Management System and through site-specific applications for Women’s and Children’s Health Network Human Research Ethics Committee on May 26, 2021, as 1 of 5 designated study sites to facilitate workshops in metropolitan and rural hospitals and health service settings in South Australia. In addition, ethical approval has been granted by the Human Research Ethics Committee at the University of South Australia on June 26, 2021 (ID: 204,074).

The participants’ identities and confidentiality were maintained throughout the research study. The participants will be informed about completing the questionnaires, attending a workshop, and conducting follow-up interviews.

In phase 1, the questionnaires will be coded to deidentify participants and linked to a participant’s identity for reidentifying purposes to allow follow-up data to be monitored and analyzed.

In phase 2, if the participants wish to participate in an interview, this will be audiotaped for further analysis. Participants who attend an interview will be deidentified, and data findings will be nonidentifiable. Nonidentifiable findings will be published in peer-reviewed journals and presented at conferences.

There is no known risk or harm, that is, physical, psychological, spiritual, emotional, social or financial well-being, or employment, from participating in this study or publicizing its results or findings. A support protocol flowchart will guide the researchers to support any participant who is identified or who shows signs of anxiety or stress, and a well-being card will be given to participants with information on how to contact counseling services and the employment assistance program.

### Informed Consent

Health professionals (nurses and midwives) will be able to access a participant information sheet (PIS) from the study website. The PIS will include information regarding the aim of the research, what the participation will involve, the workshop details, confidentiality, the consent process, and how the research information will be collected and used. The PIS will inform health professionals (midwives and nurses) that participation in the study is voluntary and that they can withdraw at any time. Participants will not be identified; they will be allocated a participant code number (ID). They can also contact the primary researcher (MS) at any time during the study for any further information. Participants will be required to give their consent and sign a consent form to participate in phases 1 and 2 of the study and to attend a *You Matter: Finding Your Self-Compassion* workshop.

### Data Management Process

The data management process will be organized according to the University of South Australia guidelines and the My Data Management Plan Tool (University of South Australia). The participants’ confidentiality will be maintained through a deidentified collection of data. The storage archiving of data is through software data stored on the web at the University of South Australia local server. Data files will be stored in at least two locations to reduce complete loss, including USB drives and personal laptops, and the data will be frequently backed up on the University of South Australia server. All soft copy data collected (questionnaires and interviews) during the study will be kept on a computer (password protected) to keep the research data confidential and limited to the research team. Hard copy data collection tools will be stored in a locked filing cabinet in a locked room in the Clinical and Health Sciences Unit, University of South Australia. Files within the folder will have a clear name so that the research team can find the related documents. These measures will be taken to ensure the security of information from misuse, loss, or unauthorized access while stored during the research project. The research data and records will be maintained for 5 years after publication. This storage of data requirements complies with the ownership and retention of data policy as outlined by the National Statement on Ethical Conduct in Human Research. Regarding secure data destruction, the primary researcher will obtain written approval from the Executive Dean of the University of South Australia Clinical and Health Science Unit for secure destruction of the research data, the materials, and associated research records. This data material will be shredded and placed in secured document destruction bins. All data stored electronically will be deleted through a process of repeated overwriting of the documents and deletion from the server, ensuring that the contents cannot be recovered.

## Results

### Overview

A scoping review was previously undertaken to inform this research study, focusing on nurses and midwives [15]. The preparatory phase was completed on April 2021. Phase 1 is expected to be completed by June 2022, and phase 2 will commence in July 2022.

### Integration of Quantitative Results and Qualitative Findings

Data integration requires an appropriate approach that knows when to combine results and investigate contradictory findings [40]. The findings from phase 1 will help develop and guide the undertaking of phase 2. The findings from phase 1 (quantitative) will be integrated and mixed with the findings from phase 2 (qualitative). This data integration will provide more in-depth and deeper insights into how self-compassion can influence the health professionals’ health and well-being. Collecting the findings and analyzing data from both phases will lead to combined outcomes to draw acceptable conclusions. The sequential explanatory design enables reporting the results from the 3 time point questionnaires, findings from the *your self-compassion* workshop, and the interviews to make general conclusions from the collective results or findings.

### Phase 3: Dissemination and Translation of Findings and Recommendation

A project report will be written for any funding bodies, and a summary of the key findings and recommendations will be uploaded and accessible via the study website. The findings and recommendations of this mixed methods study will be disseminated through publications, conference presentations, and professional and social media platforms.

## Discussion

### Principal Findings

The previously undertaken scoping review concluded that self-compassion education and training can improve the midwives’ and nurses’ awareness and increase their ability for self-compassion. It suggests that there is a clear justification to undertake this research study by exploring and investigating the influence of self-compassion on midwives and nurses.

### Conclusions

The key findings from the data integration of this research project will provide in-depth details and insights to broaden the discussion about self-compassion and its influence on health professionals’ health and well-being. Therefore, the health professionals (nurses and midwives) would benefit from developing and designing self-compassion education and training programs to improve their health and well-being.

## Appendix

Multimedia Appendix 1 External Peer-Review Report 1 by the University of South Australia, Clinical & Health Sciences.

Multimedia Appendix 2 External Peer-Review Report 2 by the University of South Australia, Clinical & Health Sciences.

## Abbreviations

PIS: participant information sheet
