# Supplementary material for: Self-compassion Education for Health Professionals (Nurses and Midwives): Protocol for a Sequential Explanatory Mixed Methods Study
Source: JMIR Res Protoc. 2022 Jan 13;11(1):e34372. doi: 10.2196/34372 (PMC8796041; doi:10.2196/34372)
Supplement: Multimedia Appendix 1 [file resprot_v11i1e34372_app1.pdf]

UNIVERSITY of BRADFORD

d.wepa@bradford.ac.uk

You Matter: Finding your self-compassion education for Health Professionals: A sequential explanatory mixed methods study protocol		
**Reviewer Responses:**	**Researcher Responses**	**Page**
**Reviewer 1: Associate Professor Dianne Wepa**		
1. Please amend and make clearer the research question or questions that the project is intended to explore".	***The Research question/s have been made clearer for participants to understand.***<br><br>2.2 Research Questions<br><br>What does self-compassion mean to health professionals **(nurses and midwives)**?<br><br>What does compassion for others mean to health professionals **(nurses and midwives)?**<br><br>What influence will self-compassion education and training have upon health professional's **(nurses and midwives)** health and wellbeing?	Research Protocol Page 4
2. The study's aim and objectives need to be written more clearly. Recommend using an overall aim for the full study and the aim for this first study re: nurses and midwives as the target population.<br><br>The study will assess whether there is an association between high levels of self-compassion, mood and wellbeing and "what impact will self-compassion education and training have upon health professionals (nurses and midwives)' health and wellbeing as a	**Aim has been amended and objectives has been added to the research protocol:**<br><br>**Overall Aim:** To increase knowledge and understanding of self-compassion and how this may enhance health and wellbeing of health professionals.<br><br>**Aim for this first study:** To increase knowledge and understanding of self-compassion and how this may enhance health and wellbeing of (nurses and midwives).<br><br>**Objectives**:<br><br>To find out and explore what self-compassion means to **nurses and midwives**<br><br>To find out what being compassionate to others means to **nurses and midwives**	Research Protocol Page 3-4

	component of self-care". Therefore, the objectives need to be in alignment with what outcomes will be assessed.<br><br>Recommend the researchers amend and make the research study aim and objectives clearer and this first study's target population will be nurses and midwives.	To find out if there is an association between self-compassion and levels of anxiety, stress, mood, wellbeing<br><br>To provide education to develop self-compassion strategies<br><br>To enhance nurses and midwives' skills for self-compassion	
3.	It is unclear whether the efficacy of the workshop will be assessed. If this is the case, please make this information clearer.	**To clarify:**<br>The self-compassion workshop has already been piloted, and evaluated, therefore, the efficacy of the workshop is not been undertaken.	Research Protocol Page 2
4.	Please provide further information re: the power calculation undertaken for this study.	**Information re: the power calculation for single group, repeated measures sample size.**<br><br>"In South Australia, there are approximately, 57,784 health practitioners registered (based on 2018/2019 registration statistics), of these (678 are midwives, 32361 nurses and 1854 have dual registration). A power calculation using a single-factor, repeated measures design, estimated a sample of 380 participants, measured at three time points, for levels of self-compassion (primary outcome) will achieve 95% power to detect differences between pre and after the education using a Geisser-Greenhouse Corrected F Test at a 0.05 significance level (P < 0.05). Therefore, the study aims to recruit 400 healthcare professionals (nurses and midwives) to account for potential loss to follow-up with the intervention requiring completion of pre, immediate and post-test educational questionnaires."	Research protocol Page 6-7

5. Please provide further information on the content of the self-compassion program and clarify whether it is evidence based.	**Further information on the content of the education workshop**<br><br>A list of the content covered is included in the protocol. The workshop is for registered nurses and midwives and not the general population, some scholarly writing is included. Yes, we can confirm that the content is evidence-informed and there is a specific section that refers to current evidence. In addition, a scoping review titled 'The influence of self-compassion upon midwives and nurses health status has been published, and the workshop facilitator/s will discuss the findings.	Research protocol Page 8
6. Researchers are responsible for the participants welfare during the study. Therefore, recommend a more proactive process to be put in place which enables the researchers to actively follow up with participants whose responses suggest that they may be experiencing distress.	**A more proactive process to support a distressed participant is now in place**, i.e., the primary researcher will actively follow up any participant whose questionnaire results suggest that they may be experiencing anxiety, stress, low mood.	
7. Additionally, Employment Assistance Program (EAP) information should be transparent in the information sheet if participants are referred to the EAP.	Please see the Support Strategy Protocol and Support Card (list support and helpline telephone numbers).<br><br>**Researchers will not refer a participant to EAP**. Therefore, information is not required on the PIS. Participants will self-refer. (participants are registered nurses/midwives and will be aware of and how to access EAP as employees of WCH.<br>The researchers will off support and signpost/guide any nurse/midwife participant to support resources and counselling services, including (EAP) if they score highly on the questionnaire, disclose or show any emotional or physical signs for anxiety and/or stress and	(separate appendix)

	will check-in and follow up the participant the following week.	
8. Please clarity who will conduct the interviews and whether this will be a member of the team who developed the self-compassion program. The interviews should be conducted by an independent person, namely, someone who was not involved in the design of the program. Please make it clear who will be undertaking the follow up interviews.	The interviews will be conducted by Professor Annette Briley and a Research Assistant (TBC). **To clarify:** No member of the team involved in the development of the educational workshop will conduct the interviews.	
