# Supplementary material for: Self-compassion Education for Health Professionals (Nurses and Midwives): Protocol for a Sequential Explanatory Mixed Methods Study
Source: JMIR Res Protoc. 2022 Jan 13;11(1):e34372. doi: 10.2196/34372 (PMC8796041; doi:10.2196/34372)
Supplement: Multimedia Appendix 2 [file resprot_v11i1e34372_app2.pdf]

UniSA | Clinical & Health Sciences

You Matter: Finding your self-compassion education for Health Professionals: A sequential explanatory mixed methods study protocol		
**Reviewer Responses:**	**Researcher Responses**	**Page**
**Reviewer 2**		
1- Recommend including in participation information sheet (PIS) a section title "What does participation in the study involve?". Information about what participation will involve should be added to the information sheet.<br><br>Additionally, include information re: the questionnaires and what type of questions will be asked, the workshop and what this will involve, what participants will be asked about re: their past history of stress and anxiety, etc.	**What does participation in the study involve?"**<br><br>A section has been added to the PIS.<br>Further information has been added to the PIS re: the questionnaire and type of questions.<br>There is an included question about your previous history of any anxiety and stress (for a baseline measurement).<br><br>Further information about attending the workshop has been added.	Research protocol Page 7
2- It is stated that a website "compassionateselfcare.org" OR .com will be designed but is not currently operational – please confirm this.	Yes, the study website will be operational once funding has been granted and when the study commences.	Research protocol Page 7
3- *The purpose of this study, it is recommended* that more information should be included. The purpose of the study will look at whether the workshop has a positive effect on self-compassion and	The objectives have been made clearer to understand and are in alignment with the overall aim of the study.<br>Pre and post educational workshop measurements will measure whether there	Research Protocol Page 3-4

whether prior anxiety and stress affects self-compassion care.	has been a positive effect on nurses and midwives' mental health and wellbeing.	
4- Recommend that the Inclusion of a section on the risks and benefits of being involved in the study. This should include information on the process for following up participants who show signs of distress and need support.	A support strategy protocol and support card (with support and counselling contact details) has been included in version 2. The support card will be given to all participants as they may be a participant who has not been identified or disclosed having high levels of anxiety and stress.	
4- Information about ethical approval<br>Please amend and include that ethical approval has been granted and by whom. The information is necessary re: should any participants/potential participants wish to discuss the approval process, or any concern or complaint.	The study has the approval of the WCHN Human Research Ethics Committee and UniSA Health and Social committee. The name and contact details of the Executive Officer and Ethic chairperson has been added to the PIS.	Research Protocol Page 10
